# The Mixed Tendency in Bipolar Disorder: An Operational Proposal for the Integration of Mixed Episodes in Predominant Polarity

**DOI:** 10.3390/jcm12237398

**Published:** 2023-11-29

**Authors:** Giovanna Fico, Gerard Anmella, Michele De Prisco, Vincenzo Oliva, Chiara Possidente, Lorenzo Bracco, Marta Bort, Tabatha Fernandez-Plaza, Anna Giménez-Palomo, Eduard Vieta, Andrea Murru

**Affiliations:** 1 Bipolar and Depressive Disorders Unit, Institute of Neuroscience, Hospital Clinic, University of Barcelona, IDIBAPS, CIBERSAM, 08036 Barcelona, Spainanmella@clinic.cat (G.A.);; 2 Department of Biomedical and Neuromotor Sciences, University of Bologna, 40126 Bologna, Italy; 3 Department of Pathophysiology and Transplantation, University of Milan, 20122 Milan, Italy

**Keywords:** bipolar disorder, predominant polarity, mixed tendency, mixed episodes, suicide, course specifier, rapid cycling

## Abstract

Predominant Polarity (PP) is an established specifier of Bipolar Disorder (BD), holding significant clinical implications. Nevertheless, there exists no consensus on how to incorporate mixed states into PP, leaving patients prone to mixed recurrences that are unclassified. In a comprehensive study involving 701 euthymic BD patients, we sought to redefine PP by introducing a novel metric, the “mixed tendency”, and establish a practical threshold to identify patients with a “mixed phenotype”. Furthermore, we investigated potential associations between the mixed phenotype and specific PP categories. Our findings revealed that the mixed tendency correlated significantly with early BD type I, lifetime suicide attempts, self-aggressive behaviour, and lifetime number of affective episodes (>5). Using a ROC curve analysis, we determined an optimal cut-off point for the mixed tendency at 0.228, suggesting that patients with ~25% of lifetime mixed episodes relative to total affective episodes should be identified as having a mixed phenotype. Notably, the mixed phenotype was positively associated with undetermined PP and negatively with manic and depressive PP. This study introduces a promising approach to incorporating mixed episodes into the PP definition, potentially enabling tailored interventions for patients with a substantial history of mixed episodes. However, further research in large, longitudinal cohorts is essential to validate these findings.

## 1. Introduction

The concept of predominant polarity was forged in the 1960s [1] and the late 1970s [2] to define subgroups of patients with bipolar disorder (BD) who mainly experience recurrences of depressive or manic episodes. Afterward, the need to operationalize the concept led to the current two most accepted definitions of the predominant polarity specifier: (1) the Barcelona proposal [3], requiring patients to present at least two-thirds (2/3) of lifetime episodes of one polarity and (2) the Harvard proposal [4], requiring patients to present a simple majority of episodes of one polarity for inclusion into either category. The Harvard proposal has less stringent criteria, finally classifying more patients within a predominant polarity but with considerably less diagnostic stability [5] and subsequent less solid clinical characterization.

According to the latest systematic reviews, a depressive predominant polarity (DPP) is found in 17.0–34.1% (median value: 21.4%) of the BD samples, whilst a manic predominant polarity (MPP) is observed in 12.4–55.0% of participants (median value: 26.0%). Predominant polarity is undetermined in 47.3% of patients with BD (range: 28.2–57.6) [6,7], so the majority of patients with BD cannot be categorized according to this concept.

Clinical correlates of affective predominance may vary depending on the polarity subtype. DPP is positively associated with the female sex, first depressive episode (or index episode) [8], later BD onset and first psychiatric hospitalization [9], higher risk of suicide [10], and a seasonal pattern [11]. MPP is positively associated with earlier age of onset [12,13], psychotic symptoms [8], higher lifetime psychiatric hospitalizations, substance abuse [13], and cognitive impairment [14].

Aside from clinical stratification within BD, predominant polarity contributes to a more specific, patient-tailored pharmacological or psychological treatment plan [8]. Indeed, the Polarity Index, calculated as the ratio between the number needed to treat (NNT) for prevention of depression and NNT for prevention of mania as emerging from the results of randomized placebo-controlled trials, is a clinically useful algorithm that reflects antimanic versus antidepressant maintenance efficacy of available treatments for BD according to predominant polarity [8,15]. Also, different predominant polarities seem to depict different neurobiological underpinnings [16] that are related to different phenotypes in BD [17].

The definition of MPP and DPP based on two-thirds of episodes [3] is the most consistently used and validated [18,19,20,21,22], but it called for different criticisms [23]. First, it implies a restrictive definition so that the highest percentage of patients, roughly 50% [6,7], end up without having a predominant polarity in the recently clinically characterized category of undetermined predominant polarity (UPP) [20]. Secondly, mixed episodes do not compute clearly into a specific polarity, being included either in MPP [24] or DPP [25]. Alternatively, mixed episodes have been counted for the total number of episodes but not for a specific polarity [3], thus contributing to inflation in the proportion of patients that do not present a predominant polarity (i.e., UPP) [20].

The diagnosis of mixed states in BD underwent a significant shift from the categorical approach in DSM-IV to the dimensional mixed features specifier in DSM-5 [26], which can be applied to both manic and depressive episodes. Although these changes reflect a more inclusive approach to capturing the complexities of BD mood presentations, substantial controversy still remains as to whether the definition proposed reflects the empirical evidence [27].

BD patients with frequent mixed episodes tend to have a more severe and chronic course of illness, often exhibiting rapid mood swings, increased aggressive behavior, irritability, treatment resistance requiring additional interventions beyond standard mood stabilizers, comorbid psychiatric disorders, and cognitive impairment [28,29,30]. Proper diagnosis, comprehensive assessment, and individualized treatment planning are essential for managing BD patients with frequent mixed episodes [31,32].

The present study primarily aims to explore the integration of mixed episodes in the concept of predominant polarity in BD by defining a new quantitative measure, the “mixed tendency”, and proposing an operational cut-off for clinical implementation: the “mixed phenotype”. Secondarily, the study seeks to understand whether patients with a specific predominant polarity are more susceptible to experiencing mixed episodes.

By developing this operational proposal, healthcare professionals might be provided with a precise and objective tool to identify and characterize patients with a mixed phenotype, which can guide through treatment selection and improve clinical outcomes.

## 2. Materials and Methods

We conducted a naturalistic study including cross-sectional data derived from a prospective cohort of patients.

### 2.1. Sample and Inclusion/Exclusion Criteria

All consecutive patients admitted to the Bipolar and Depressive Disorders Unit of the Hospital Clinic of Barcelona from October 2000 to May 2021 were considered for inclusion in the present study. Inclusion criteria were: diagnosis of BD type I (BD-I) or II (BD-II), age above 18, and euthymia [33]. Diagnoses were consistently assessed by trained psychiatrists using the Structured Clinical Interview for DSM-IV-TR [34] and DSM-5 [35]. The main socio-demographic and clinical characteristics were collected through a semi-structured interview with the patient and caregivers, including the number and polarity of previous episodes, hospitalizations, age of onset, presence of psychotic symptoms, suicidal and non-suicidal self-injurious behavior, hetero-aggressive behavior, and comorbid psychiatric diagnoses, as well as current (at least 3 months duration) and past pharmacological treatments. Exclusion criteria were severe cognitive, motor, or visual impairment or severe medical conditions requiring immediate hospitalization at baseline assessment. All participants provided written informed consent for this ethical committee-approved study.

### 2.2. Predominant Polarity and Assessment of Mixed Episodes

We assigned a predominant polarity according to the Barcelona proposal [3], requiring participants to have at least two-thirds of lifetime affective episodes of one polarity in order to be included in either the MPP or DPP groups. Patients with a unique episode were assigned a predominant polarity equal to the polarity of the index episode (MPP), according to both the Barcelona proposal criteria and the known value of the onset episode to predict the polarity of subsequent episodes over time [36]. Patients who could not be categorized as either DPP or MPP were included in the UPP category.

### 2.3. Defining the Mixed Tendency and Mixed Phenotype

Mixed episodes were included in the total episode count but not for a specific predominant polarity. Mixed episodes diagnosed with DSM-IV-TR (before 2013) or depressive, manic, or hypomanic episodes having mixed features according to DSM-5 criteria were recorded as “mixed episodes”. We created a new quantitative measure, the “mixed tendency”, a continuous variable calculated as the ratio of mixed episodes to total affective episodes for each participant. We hypothesized that the clinical variables associated with the mixed tendency would allow us to identify patients with a “mixed phenotype” (i.e., mixed patients) and discriminate them from patients without it (i.e., non-mixed patients).

### 2.4. Statistical Analysis

The Kolmogorov–Smirnov test was used to assess whether continuous variables displayed a normal distribution. Spearman’s correlation or Mann–Whitney U tests were used to test the association between the mixed tendency and continuous and categorical clinical variables, respectively. Individuals were excluded from the analysis if they presented more than 50% of the missing variables. Variables that reached statistical significance were used to compute a new dichotomous variable named “mixed phenotype” (1 = at least one of the significant variables was present in the same patient, 0 = all the other cases). Continuous significant variables were dichotomized for defining patients with the mixed phenotype. To assess the predictive ability of the mixed tendency for identifying the mixed phenotype, a ROC curve analysis was conducted on patients presenting at least one lifetime mixed episode. The ROC curve was constructed by plotting the sensitivity (true positive rate) against 1-specificity (false positive rate) at different threshold values of the mixed tendency. The area under the curve (AUC) was calculated as a measure of the overall discriminatory power of the mixed tendency in predicting the mixed phenotype. The AUC ranges from 0.5 (no predictive ability) to 1.0 (perfect discrimination). The optimal cut-off point on the ROC curve was determined based on Youden’s Index, which maximizes the sum of sensitivity and specificity minus one. The cut-off point corresponding to the maximum Youden’s Index was selected as the threshold value for defining the mixed phenotype based on the mixed tendency. The performance of the cut-off point was assessed using sensitivity, specificity, and accuracy. Sensitivity measures the proportion of correctly identified patients with the mixed phenotype (mixed patients), while specificity measures the proportion of correctly identified patients without it (non-mixed patients). Accuracy represents the overall proportion of correctly classified patients. After establishing the optimal cutoff on the mixed tendency for defining the mixed phenotype, we assessed its association with a specific predominant polarity using a chi-square test. Statistical analyses were performed using RStudio (RStudio: Integrated Development for R. RStudio, 2020) [37]. The ROC curve analysis was conducted using the pROC package functions in the R software 4.3.2. [37]. Statistical significance was set at 0.05 and corrected for multiple comparisons with Bonferroni correction.

## 3. Results

### 3.1. Descriptive Results

The sample was composed of 701 patients, 316 men (45.1%) and 385 women (54.9%), with a mean age of 45.25 years (±14.18) and a diagnosis of BD-I (*n* = 501; 71.5%) or BD-II (*n* = 200; 28.5%). The mean number of lifetime affective episodes was 15.2 (±19.1); 15 patients (2.1%) had only one episode. The mean number of lifetime mixed episodes was 0.63 (±1.8). DPP, MPP, and UPP were present, respectively, in the (*n* = 132, 18.8%), (*n* = 135, 19.3%), and (*n* = 434, 61.9%) of the sample. The mean mixed tendency was 0.04 (± 0.1). All the other socio-demographical and clinical characteristics of the sample are reported elsewhere [20]. Patients with at least one mixed episode were 157 (22.4%).

### 3.2. The “Mixed Tendency” and Other Variables Related to the “Mixed Phenotype”

The mixed tendency was significantly associated with type I BD (z = −4.87, *p* = 1.09 × 10^−6^), suicide attempts (z = −5.83, *p* = 5.61 × 10^−9^), self-aggressive behavior (z = −3.41, *p* = 6.5 × 10^−4^), and number of affective episodes (≥5) (ref) (z = −3.71, *p* = 2.1 × 10^−4^). Patients presenting at least one of the former clinical characteristics were defined as presenting the “mixed phenotype”. Younger age of onset (years) (rho = −0.86, *p* = 0.023), rapid cycling (z = −2.31, *p* = 0.021), and psychotic symptoms during a manic episode (z = −2.07, *p* = 0.038) were significantly associated with the mixed tendency but did not maintain statistical significance after the Bonferroni correction for multiple comparisons. Thus, they were not included in defining the “mixed phenotype”.

Other clinical variables did not show a statistically significant association with the mixed tendency, including age, sex, illness duration, number of previous manic or depressive episodes, number of psychiatric admissions, non-suicidal, hetero-aggressive behavior, presence of psychotic episodes at the first affective episode, seasonality, presenting symptoms atypical or melancholic depression, or catatonia, psychiatric and non-psychiatric comorbidities, psychiatric family history, and family history of suicide.

Quetiapine treatment was associated with the mixed tendency (z = −3.83, *p* = 1.27 × 10^−4^).

Other lifetime treatments were significantly associated with the mixed tendency but did not maintain statistical significance after the Bonferroni correction for multiple comparisons. Those included clozapine (z = −2.19, *p* = 0.029), aripiprazole (z = −2.37, *p* = 0.018), asenapine (z = −2.53, *p* = 0.011), and electroconvulsive therapy (ECT) (z = −2.65, *p* = 0.008) and not receiving serotonin and norepinephrine reuptake inhibitors (SNRI) (z = −3.09, *p* = 0.002).

Lifetime treatments not associated with the mixed tendency included lithium, valproate, lamotrigine, carbamazepine, oxcarbazepine, topiramate, Selective serotonin reuptake inhibitors (SSRIs), tricyclic antidepressants, Monoamine oxidase inhibitors (MAOIs), SSRI, risperidone, paliperidone, olanzapine, ziprasidone, amisulpride, tiroxine, gabapentin, or anxiolytics.

**Figure 1:**
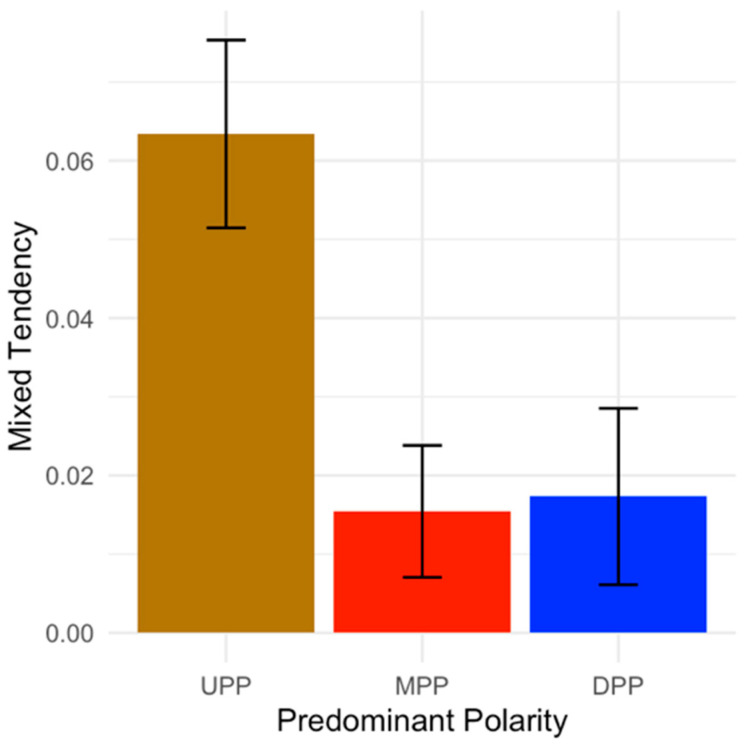
Bar plot of the Mixed Tendency (Y-axis) according to the Predominant Polarity.

The mixed tendency was significantly lower in patients with DPP (z = −3.88, *p* = 1.03 × 10^−4^) and MPP (z = −3.81, *p* = 1.37 × 10^−4^), and higher in patients with UPP (z = −6.22, *p* = 4.87 × 10^−10^) (Figure 1).

### 3.3. The Mixed Phenotype: Calculating the Optimal Mixed Tendency Threshold

**Figure 2:**
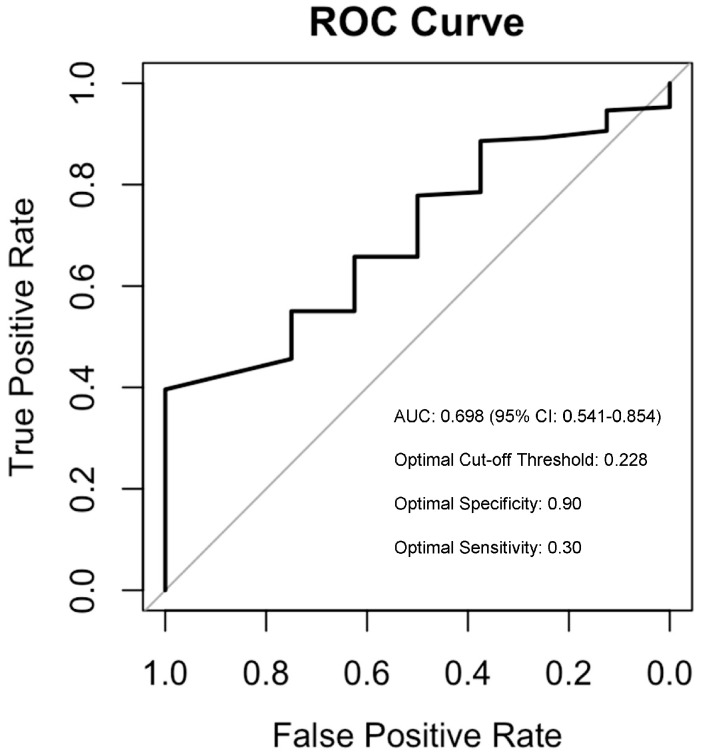
ROC Curve for predicting patients with Mixed Phenotype.

The ROC curve (Figure 2) illustrates the trade-off between sensitivity and specificity across different threshold values of the mixed tendency for defining patients with the mixed phenotype.

The area under the curve (AUC) was 0.698 (95% confidence interval [CI]: 0.541–0.854), indicating a fair discriminatory power of the mixed tendency in identifying patients with the mixed phenotype. The optimal cut-off point of the mixed tendency on the ROC curve was found to be 0.228, suggesting that patients with more than ~25% (1/4) of lifetime mixed episodes of the total number of affective episodes should be considered as having a mixed phenotype. This cut-off yielded an optimal sensitivity of 0.66 and a specificity of 0.63.

### 3.4. The Mixed Phenotype within Predominant Polarities

**Figure 3:**
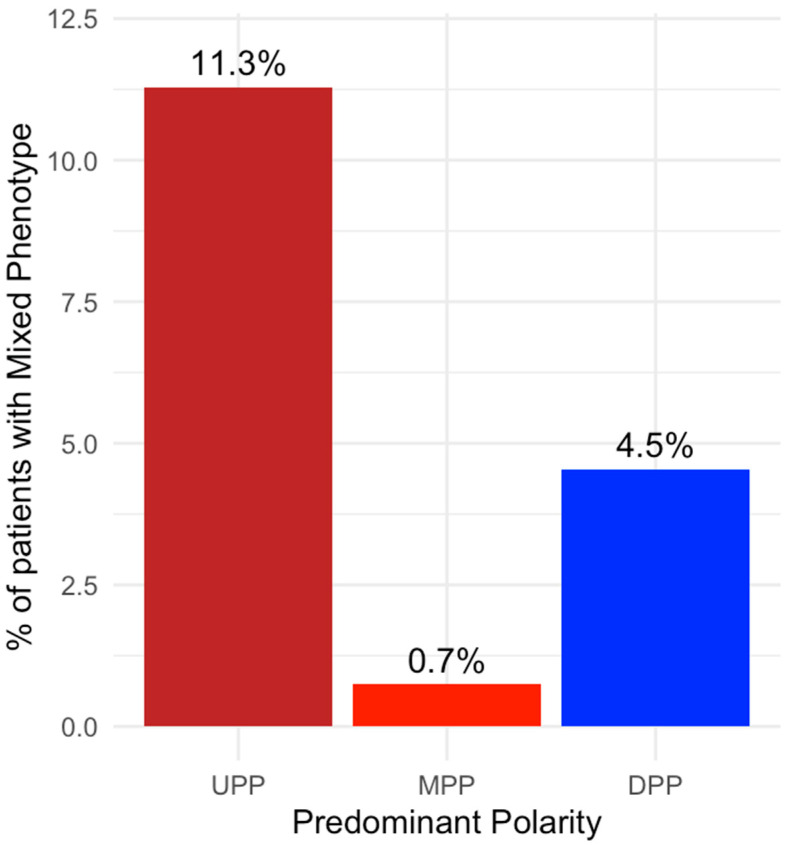
Proportion of individuals with mixed phenotype according to predominant polarity. X-axis: Undetermined (UPP), Manic (MPP), and Depressive (DPP) Predominant Polarity. Y-axis: Proportion of patients with the “Mixed Phenotype” according to the calculated cut-off.

Patients were categorized into two groups according to the 0.228 mixed tendency cut-off point (with and without the mixed phenotype). The mixed phenotype was present in 49 (11.3%) individuals with UPP, 1 (0.7%) with MPP, and 6 (4.5%) with DPP. The mixed phenotype showed a positive significant association with UPP (χ^2^ = 15.74, *p* = 7.3 × 10^−5^) and a negative significant association with DPP (χ^2^ = 10.76, *p* = 1.0 × 10^−3^) and MPP (odds ratio = 0.07, 95% CI 0.002–0.41, *p* = 1.2 × 10^−4^), with significant differences between the three groups (χ^2^= 18.21, df = 2, *p* = 1.1 × 10^−4^) (Figure 3).

## 4. Discussion

In our study, we performed a novel approach with the aim of integrating mixed episodes into the framework of predominant polarity. The mixed tendency is related to a specific phenotype among patients with BD, characterized by type I BD, a history of suicide, self-aggressive behaviors, and a higher number of affective episodes. These clinical characteristics have been found to be associated in several studies with patients with index mixed episodes or presenting mixed features during acute affective episodes [38,39,40]. Moreover, we assessed the capacity of the mixed tendency to identify patients with the mixed phenotype by deriving an optimal cut-off from the ratio of lifetime mixed episodes over total affective episodes. The proposed optimal cut-off was 0.228, suggesting that patients with more than 22.8% (approximatively 1 out of 4) of lifetime mixed episodes of the total number of affective episodes were more likely to present the mixed phenotype. This corresponds to 7.9% (56/701) of our total sample or 35.7% (56/157) of patients with at least one mixed episode during their lifetime. Patients with the mixed phenotype might underpin a different subpopulation of patients with specific clinical characteristics. The fact that the optimal cut-off of the ROC curve is relatively small means that the model prioritizes sensitivity over specificity. Thus, it will correctly identify a higher proportion of individuals with the mixed phenotype but may also misclassify some individuals without the phenotype. Since the introduction of the “mixed features specifier” in the DSM-5, several criticisms have been raised. In a recent review, the ranges of hypo/manic episodes with mixed features (4.3–58.6%) or depressive episodes with mixed features (0–34%) were found to be low, possibly reflecting the low sensitivity of the “mixed features specifier” [40].

This change in the criteria has raised concerns that the revised definition may not capture the full complexity and richness of the mixed-state presentation. By omitting symptoms like psychomotor agitation, irritability, and distractibility, there is a risk of overlooking important diagnostic features and potentially misclassifying individuals who exhibit mixed features [41].

According to these premises, we believe that the computation of predominant polarity, either excluding affective episodes with mixed features or including them in a specific polarity, may exclude a complex population of patients, preventing tailored, effective interventions.

The results of our study reveal significant associations between the mixed phenotype and different predominant polarities. The positive association between the mixed phenotype and UPP is expected and supported by previous research since mixed episodes are included in the total number of affective episodes when calculating the predominant polarity in most studies [42,43], thus accounting for an increase in UPP representation in the sample. Furthermore, UPP has been associated with an increased number of lifetime mixed and total affective episodes, seasonality, and aggressive behaviors [20], similar to the mixed phenotype.

On the other hand, our results indicate a negative association between the mixed phenotype and DPP and MPP, which highlights the distinct characteristics and clinical implications of mixed states within BD. Indeed, previous evidence on the relationship between a specific polarity and mixed-states is somewhat contrasting. Mixed states and having a mixed onset episode have been associated with DPP in multisite-sample studies [4]. Also, a higher suicidal risk is linked with DPP, which indirectly links to mixicity [10,44]. Consequently, when mixed-states are added to a broader category of “depression-plus-mixed” predominant polarity, it almost doubled the risk of suicidal acts from 2.4- to 4.5-fold excess over manic PP, and suicidal risk was associated continuously with increasing proportions of depressive or mixed episodes [4]. Conversely, another study showed higher levels of mixed features during the depressive episodes of patients with MPP [45]. Also, the number of previous hypomanic episodes was found to predict the occurrence of mixed depressive episodes [46]. Including mixed episodes in a specific predominant polarity could dilute the effect of the presence of these episodes on patients’ phenotypes.

Predominant polarity serves as a valid long-term course specifier in BD, bearing significant therapeutic and prognostic implications, such as the polarity index. When conducting clinical evaluations, it is crucial to include an assessment of mixed episodes both retrospectively and during prospective follow-up. Particularly for individuals who have experienced a substantial proportion of mixed episodes (approximately more than one-seventh of total episodes), it becomes even more critical to consider prophylactic treatment strategies that extend beyond preventing manic or depressive relapses. A specific focus should be directed toward addressing the risk of mixed recurrences and mitigating adverse outcomes, including suicidal behavior. Our proposal using the mixed tendency as an operational parameter aims to help clinicians identify this high-risk population while also better characterizing a broad spectrum of the BD, which has so far fallen under the vague category of UPP.

**Figure 4:**
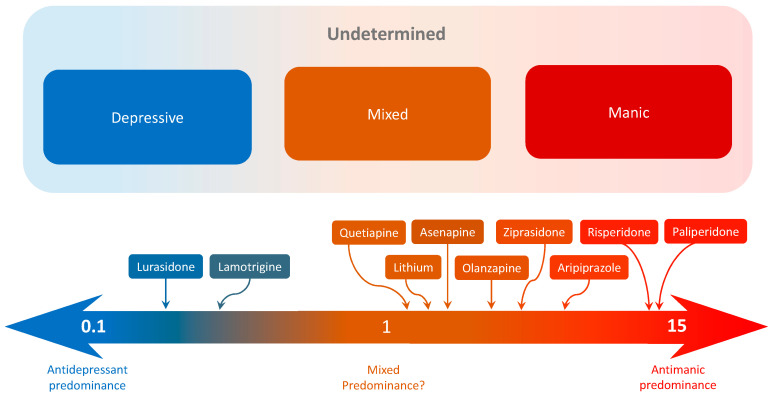
Graphical representation of the interaction between predominant polarities, the mixed phenotype, and the polarity index. In the upper part, the mixed phenotype is mostly represented by individuals with Undetermined Predominant Polarity, compared with a minority of individuals with Depressive or Manic predominant Polarity. At the bottom, the arrow represents the polarity index of different drugs, with an antidepressive predominance (on the left) or antimanic predominance (on the right). Intermediate phenotypes might benefit from drugs with a polarity index closer to 1. Note that most of the drugs with intermediate phenotypes have positive evidence for efficacy in patients with mixed episodes and may also be beneficial for patients with a mixed phenotype.

According to recent guidelines [40,47], potential treatment options for patients with a mixed phenotype involve the use of atypical antipsychotics during acute episodes (e.g., quetiapine) or a combination of mood stabilizers (e.g., lithium or valproate) and atypical antipsychotics during the maintenance phase. In this context, it can be inferred that medications with a polarity index closer to 1 might be beneficial for treating patients with a mixed phenotype, as can be seen in Figure 4. Indeed, in our study, patients presenting more mixed episodes (higher mixed tendency) were more likely to receive quetiapine. As can be seen in Figure 4, quetiapine is the pharmacological treatment with a polarity index closer to 1, which reflects a lack of antidepressant or antimanic predominance. However, it is important for longitudinal studies to validate this hypothesis, and the polarity index of newly approved drugs for BD treatment should be calculated based on randomized clinical trials to enable a more individualized approach to patient treatment.

## 5. Conclusions

In conclusion, this study provides valuable insights into the relationship between predominant polarity and mixed episodes in BD and proposes a potential integration of mixed episodes in the definition of predominant polarity. By considering mixed episodes in the definition of predominant polarity, we expand the conceptual framework and enhance our understanding of this complex condition. The mixed tendency might be a valid operational parameter to define a subpopulation of patients with BD. Patients presenting more than one-quarter of lifetime mixed episodes show differential clinical characteristics that might require tailored interventions. Our findings highlight the need for further research with larger longitudinal samples and more patient variability to delve deeper into these associations and elucidate specific clinical characteristics that may help identify individuals prone to experience more mixed episodes throughout the course of their illness.

## Data Availability

Data are available upon request.
